# Determination of safe zone for median nerve in carpal tunnel using anatomical landmarks

**DOI:** 10.6026/973206300213130

**Published:** 2025-09-30

**Authors:** Khizer Hussain Afroze M., Sangeeta M., Kumaraswamy Revanakimath, Umesh S.N., Dakshya Perumal

**Affiliations:** 1 Department of Anatomy, MVJ Medical College and Research Hospital, Hoskote, Bangalore, Karnataka, India; 2 Department of Anatomy, Sri Chamudeshwari Medical College Hospital and Research Institute, Channapatna, Karnataka, India

**Keywords:** Palmaris longus, anatomical landmark, radial styloid process

## Abstract

Carpal tunnel release, though common, carries a risk of iatrogenic median nerve injury, making it important to determine its safe zone within the tunnel using consistent palpable. In this study, 40 formalin-fixed upper limbs were dissected, and distances from the radial styloid process (RSP), palmaris longus tendon (PLT), and the medial-most point of the lower end of the ulna (LBU) to the median nerve (MN) were measured. The MN was consistently medial to the PLT and deep to the flexor retinaculum, with a mean RSP-MN distance of 28.48 mm, showing significant side differences (p = 0.001). A positive correlation between RSP-PLT and RSP-MN distances (r = 0.410, p = 0.016) confirmed the PLT as a useful landmark, while RSP and LBU served as reliable alternatives when PLT was absent. Thus, we show the clinical value of these anatomical landmarks in minimizing nerve injury during carpal tunnel procedures.

## Background:

The median nerve formed by lateral and medial root, is one of the terminal branches of brachial plexus. Variations in its formation and relation to surrounding structures have been observed in recent studies [1]. The median nerve and flexor tendons pass through the carpal tunnel (CT) [2]. Carpal tunnel syndrome (CTS) is caused by compression of median nerve (MN) at the carpal tunnel. Few risk factors for CTS include sex, jobs which require increased wrist movement, rheumatoid arthritis, obesity [3]. Early diagnosis of CTS can be treated conservatively by wrist splinting, non-steroidal anti-inflammatory drugs and steroids. There is little success and high chance of recurrence in conservative treatment hence surgical interventions are done [4]. Carpal tunnel decompression is the surgery of choice which is mainly of two types: open surgery and endoscopic carpal tunnel release (ECTR) [5]. The surgery focuses on cutting the carpal tunnel ligament (CTL) which releases the pressure on MN and relieves the symptoms. CT surgery is considered as a safe procedure but there are chances of complications [6]. Direct injury to MN is a dreaded complication as it can cause immense pain and even permanent deformity of the hand. Accidental injuries can be prevented by having knowledge of the safe zone of median nerve [7]. Therefore, it is of interest to determine the safe zone of the median nerve in the carpal tunnel as well as to analyse the distance from the bony prominences and other landmarks of the median nerve in the south Indian population.

## Material and Methods:

This observational study consists of 40 disarticulated upper limbs of unknown sex with no previous history of trauma, surgery, or anomaly of the forearm and hand region were obtained from Department of Anatomy, MVJ Medical College, Bangalore. Since it was a cadaveric study, ethical clearance was exempted from our institution. The dissection was carried out by Cunningham's Manual. Distal forearm and wrist of specimen were dissected. The following parameters were measured by using digital vernier calliper (0-150 mm, 0.01 mm resolution).

[1] Distance between radial styloid process (RSP) and lower border of ulna (LBU)

[2] Distance between RSP and palmaris longus tendon (PLT)

[3] Distance between RSP and MN

[4] Diameter of MN

## Statistical analysis:

The data were measured and entered in MS Excel 2019. The data were analysed by using statistical software SPSS version 16. Continuous variables between the two groups were analysed by using student 't' test. Categorical data was analysed by using Chi-square test. The Pearson correlation coefficient was analyzed to find the association between distance to RSP-MN and distance to RSP-PLT.

## Results:

In this research, 40 specimens were utilized; however measurements were taken from 35, while 5 were excluded. Among the 5 hands excluded, 2 exhibited a bifid median nerve, the PLT was damaged in 2 specimens and it was absent in one. The distance of RSP to MN was calculated and expressed as percentage of distance between RSP to LBU. The majority of specimens were situated within the 61-70% range of the RSP to LBU distance, comprising 51.4%. The second-largest group falls within the 51-60% range, representing 31.4% (Table 2 - see PDF). The current findings (Table 1 - see PDF) indicate a strong correlation between the distance from RSP to MN and the distance from RSP to PLT (r = 0.410, p value = 0.016). This suggests that an increase in the distance from RSP to PLT is associated with a corresponding increase in the distance from RSP to MN (Figure 1 - see PDF). The mean and standard deviation of distance between RSP and LBU is 44.97 ± 4.15 mm for both hands. All parameters are slightly higher in right than left side without any statistical significance except the RSP and MN length (Table 1 - see PDF). There is emphasis on the fact that RSP and MN are different measurements among the hands. The inference from this data is that only the length from RSP to MN is different and all other measurements are same in both hands.

## Discussion:

Carpal Tunnel Syndrome (CTS) is a prevalent neuropathy characterized by compression of the median nerve (MN) within the carpal tunnel. While conservative treatments such as splinting, non-steroidal anti-inflammatory drugs and corticosteroids-can offer temporary relief in early stages, persistent or severe symptoms often necessitate surgical decompression [4]. In these cases, a thorough understanding of the median nerve's anatomy and its relationship to surface and bony landmarks becomes critical to avoid iatrogenic injury. This underscores the concept of a "safe zone" during carpal tunnel release procedures. Defining a safe surgical zone in the carpal tunnel is vital due to the close proximity of critical neurovascular structures. Inadvertent transection or irritation of the median nerve during decompression can lead to chronic pain, neuroma formation, or permanent motor-sensory deficits. As Ozcanli *et al.* highlighted, clear anatomical guidelines and reproducible surface landmarks are essential in minimizing intraoperative complications [8]. Their cadaveric study identified a rectangular "safe zone" bordered proximally by the distal wrist crease and laterally by the third webspace, which can be used during open carpal tunnel surgery. Importantly, they emphasized that deviations from the central longitudinal axis can place the nerve at risk. Our study aligns with these principles and offers quantitative data using the RSP and LBU as surface landmarks. The use of easily palpable bony landmarks ensures clinical applicability even in resource-limited surgical environments where intraoperative imaging or endoscopic guidance may not be available. A study by Sarraf *et al.* reported a significant disparity in CSA measurements at the wrist between CTS patients and healthy individuals, identifying 0.543 cm^2^ (5.43 mm^2^) as a reliable diagnostic threshold for CTS using ultrasonographic techniques [9]. Similarly, Mohammadi *et al.* provided a comprehensive comparison of the CSA at the carpal tunnel's entrance and exit, demonstrating significantly enlarged nerve dimensions in CTS-affected wrists. Their findings showed a CSA of 11.4 ± 1.7 mm^2^ at the tunnel entrance and 9.9 ± 1.2 mm^2^ at the exit in symptomatic cases, whereas the corresponding values in healthy wrists were 5.78 ± 0.9 mm^2^ and 4.7 ± 0.7 mm^2^, respectively (p < 0.001). Based on these observations, they proposed a cut-off value of 8.5 mm^2^ for diagnostic consideration at both entry and exit points of the tunnel [10]. In our cadaveric study, we measured the median nerve's mean diameter as 4.71 ± 0.98 mm (left: 4.80 ± 0.71 mm; right: 4.67 ± 1.11 mm) and found no significant side-to-side difference, thus indicating absence of lateral dominance in nerve structural dimensions despite slight unilateral variation.

Although our measurements were obtained using digital calipers rather than imaging-based CSA estimation, the observed diameters fall within the range reported for healthy individuals in prior sonographic studies. While direct conversion between diameter and CSA is not precise due to shape variability, these values can still provide a useful anatomical reference when evaluating nerve size in both clinical and surgical contexts. Moreover, maintaining an awareness of such normative data is essential for recognizing pathological enlargement in CTS and ensuring safe and accurate surgical intervention. According to the study done by Ajayi *et al.* [2], the mean distance between the RSP and USP was 49.34 mm, whereas the study conducted by Soumya *et al.* found that the distance between RSP and MMPU is 48.598 mm. In the current study, mean distance between the RSP and lower border of ulna (LBU) is 44.97mm. The mean distance of MN from RSP according to the Ajayi *et al.* was 22.44 mm while another study by Soumya, the distance of MN from RSP was found to be 22.39 mm. In the present study, the mean distance of MN from RSP was 28.48mm. It was found right side value (29.77 ± 2.96mm) is more than that left side value (26.01 ± 3.10mm) which is statistically significant. While cadaveric dissection provides accurate morphometric data, ultrasound offers a non-invasive tool for surgical planning. Chern *et al.* [11] combined ultrasonography and anatomical analysis to define safe zones for percutaneous carpal tunnel release. They found the median nerve shifts from the ulnar side at the inlet to a more central position distally, recommending radial instrument insertion to prevent neurovascular injury. This aligns with our finding that the nerve lies medial to the PLT and deep to the flexor retinaculum. Real-time ultrasound helps visualize vital structures, making a combined static-dynamic approach valuable, especially in complex or repeat surgeries. We used RSP and the LBU as landmarks, as they form a reliable anterior-posterior axis easily palpable on the volar wrist surface-more suitable than the ulnar styloid for carpal tunnel approaches. A significant correlation (r = 0.410, p = 0.016) between RSP-PLT and RSP-MN distances suggest the PLT can be a helpful soft-tissue marker. Even when PLT is absent, bony landmarks alone offer dependable intraoperative guidance. In our analysis, the mean distance from RSP to MN was 28.48 mm, but notably higher on the right (29.77 ± 2.96 mm) than on the left (26.01 ± 3.10 mm), a statistically significant finding (p = 0.001). This difference emphasizes the importance of individual preoperative assessment rather than assuming anatomical symmetry. While our study does not address hand dominance, it suggests that even subtle differences in wrist anatomy could influence the location of vital structures. Moreover, these differences hold important surgical relevance. A surgeon performing a bilateral release cannot assume that the same skin incision or retractor angle used on one hand will be safe for the other. Such an approach would increase the risk of injury to the MN, especially if accompanied by anatomical variations such as a bifid median nerve or persistent median artery. One of the novel contributions of this study lies in expressing the location of the median nerve as a percentage of the RSP-LBU distance. We found that 51.4% of specimens had the MN located between 61-70% of this distance and 31.4% fell within the 51-60% range (Table 3 - see PDF). In contrast, earlier studies by Ajayi *et al.* [2] and Soumya *et al.* [12] indicated a majority of MN locations falling under <50%, suggesting population-specific anatomical variation. Ajayi *et al.* [2] reported a safe zone located at 57.5%, while Soumya *et al.* [12] found the zone at 64.57%. Our current study noted the safe zone at 63%, a finding that is both consistent with previous literature and supportive of the use of RSP and LBU as reliable landmarks. The slightly more proximal placement in other populations may be attributed to racial, ethnic, or morphometric differences, as suggested in comparative anatomical studies [1, 2].

## Conclusion:

The median nerve was consistently located medial to the palmaris longus tendon (PLT) and deep to the flexor retinaculum, with predictable relationships to bony landmarks such as the radial styloid process (RSP) and lower end of the ulna (LBU), and showed significant side-specific variation in RSP-MN distance. A positive correlation between RSP-PLT and RSP-MN distances highlight the PLT as a reliable soft-tissue marker when present, while RSP and LBU serve as dependable alternatives in its absence. Thus, we show that the safe surgical zone lies beyond 63% of the distance between RSP and LBU, helping reduce the risk of iatrogenic median nerve injury during carpal tunnel release.
